# Role of 5-HTTLPR Polymorphism in the Development of the Inward/Outward Personality Organization: A Genetic Association Study

**DOI:** 10.1371/journal.pone.0082192

**Published:** 2013-12-16

**Authors:** Bernardo Nardi, Alessandra Marini, Chiara Turchi, Emidio Arimatea, Adriano Tagliabracci, Cesario Bellantuono

**Affiliations:** 1 Psychiatric Unit, Department of Experimental and Clinical Medicine, Polytechnic University of Marche, Ancona, Italy; 2 Section of Legal Medicine, Department of Biomedical Sciences and Public Health, Polytechnic University of Marche, Ancona, Italy; University of Medicine & Dentistry of NJ - New Jersey Medical School, United States of America

## Abstract

Reciprocity with primary caregivers affects subjects' adaptive abilities toward the construction of the most useful personal meaning organization (PMO) with respect to their developmental environment. Within cognitive theory the post-rationalist approach has outlined two basic categories of identity construction and of regulation of cognitive and emotional processes: the Outward and the Inward PMO. The presence of different, consistent clinical patterns in Inward and Outward subjects is paralleled by differences in cerebral activation during emotional tasks on fMRI and by different expression of some polymorphisms in serotonin pathways. Since several lines of evidence support a role for the 5-HTTLPR polymorphism in mediating individual susceptibility to environmental emotional stimuli, this study was conducted to investigate its influence in the development of the Inward/Outward PMO.

PMO was assessed and the 5-HTTLPR polymorphism investigated in 124 healthy subjects who were subdivided into an Inward (n = 52) and an Outward (n = 72) group.

Case-control comparisons of short allele (S) frequencies showed significant differences between Inwards and Outwards (p = 0.036, χ2 test; p = 0.026, exact test). Genotype frequencies were not significantly different although values slightly exceeded p≤0.05 (p = 0.056, χ2 test; p = 0.059, exact test). Analysis of the 5-HTTLPR genotypes according to the recessive inheritance model showed that the S/S genotype increased the likelihood of developing an Outward PMO (p = 0.0178, χ2 test; p = 0.0143, exact test; OR = 3.43, CI (95%) = 1.188–9.925). A logistic regression analysis confirmed the association between short allele and S/S genotypes with the Outward PMO also when gender and age were considered. However none of the differences remained significant after correction for multiple testing, even though using the recessive model they approach significance.

Overall our data seem to suggest a putative genetic basis for interindividual differences in PMO development.

## Introduction

A highly distinctive feature of consciousness is the construction of a unified sense of self, i.e. of a personal meaning. Within cognitive theory, the post-rationalist approach has attempted to understand the processes of individual meaning construction [1], [2], [3]. The openness and plasticity of developmental pathways underpin a predisposition to change, where learning can act by remodeling, reconstructing, and redefining the neural texture within a complex system whose fundamental characteristic is that it has an own "organization" [4], [5]. There exist “core organizing processes” at the base of every psychological experience, be it physiological or pathological. Furthermore, the conceptualization of “Personal Meaning Organization” (PMO) individuates “the specific arrangement of personal meaning processes by which each individual is provided with a sense of oneness and historical continuity in the course of his/her lifespan” [1]. Among behavioral patterns, the attachment relationship plays a key role in the adaptation process and allows to modulate the intensity, duration and frequency of emotional states, to orient sensory-perceptual and motor activities, and to guide in the organization of the individual's cognitive and emotional inventory [6], [7], [8]. The attachment behavioral system selects the child's adaptive abilities that are useful to achieve or maintain close proximity to the care-giver. The constancy and predictability of care-giver behaviors and emotional expressions enable early decoding of similar activations in the child, and synchronization of their psycho-physiological rhythms. As a result, the child reads the situations taking place in his environment through his own internal activations (“Inward” focus). Inward subjects therefore use bodily reactions primarily to read the environment as dangerous/available; basic emotions such as fear and sadness and their control have a pivotal role in regulating the emotional life of Inward subjects [9], [10], [11], [12], [13].

Functional neuroimaging (fMRI) studies have documented a degree of interindividual variability in the neural networks of emotion that characterize Inward/Outward subjects. Thus "phobic-prone" individuals (an Inward dimension) engage the amygdala to a greater extent than "eating-prone" subjects (an Outward dimension) during perceptual processing of threatening stimuli [14] and recruit greater neuronal resources in the medial prefrontal cortex (mPFC) during cognitive labeling of threatening facial expressions [15]; moreover they demonstrated a greater recruitment of the posterior insula, which is involved in internal bodily and subjective feeling states [16].

In contrast, when care-giver behaviors and expressions are perceived as being not thoroughly predictable, depending on external requirements, expectations and rules which the child is as yet unable to decipher, they are found to be more difficult to decode. In these cases the child needs to read the environmental signals, while emotional activations require him/her to use self-evaluating cognitive schemata. In such conditions the subject's self develops, moving from a preliminary evaluation of environmental messages that drive recognition of internal activations and self-perception (“Outward” focus) [9], [10], [11], [12], [13].

Outward subjects learn to read information from their significant environment to update their internal perceptions in terms of acceptance/refusal, high/low agreeableness and self-importance/insignificance [12], they thus build inner stability by referring to the outside world and attempt to match their own emotions with it [15].

Consequently an ability to cope with specific stress categories—relating to physical danger or loneliness in the Inward PMO and to semantic judgment and duty in the Outward PMO—is an adaptive competence in both types of PMOs.

The main clinical features related to the Inward/Outward experience focus are summarized in Table 1 [11]. Furthermore, although the two PMO patterns can coexist and are more or less evident in different subjects, one pattern prevails, at least where specific categories of experience are concerned [13].

**Table 1:** Main clinical features of the Inward or Outward experience focus [11].

Main Clinical Features	Inward Subjects	Outward Subjects
Perception of care-giver attitude (attachment)	Predictable and recursive, centred on the sureness/dangerousness environmental	Less predictable and changing, centred on environmental requests or rules
Stable patterns	Use of internal activations (i.e., fear, rage) to read environment’s characteristics (i.e., if it is available, dangerous, etc.)	Reading of external messages to realize internal adequacy or normality (i.e., if one is normal or not, good or bad, keep up with something or not, etc.)
Emotional activations	Prevalence of basic feelings (fear, rage, sadness, happiness)	Prevalence of emotional schemata (shame, blame, niceness, etc.)
Cognitive abilities	Pointed to practical and key aspects of life (evaluating dangerous changes, help availability and accessible coping abilities)	Pointed to other thoughts and expectations, social rules, etc. (how to perform personal goals according to external requests and internal criteria)
Reciprocity construction	Based on perception of physical distance from others (i.e., their presence or absence, goodwill or hostility)	Based on perception of semantic significance of environmental messages (i.e., as parameter of one’s own personal attitude toward others or of intrinsic self value)
Environmental control	Adaptation ability in performing protection and availability of others, on the one hand, and loneliness and abandonment on the other	Adaptation ability in reaching approval and agreement, on one hand, and focus certainties, good rules and values on the other

The attachment system has a well-known role in PMO development and the fMRI data mentioned above suggest a degree of interindividual variability in emotions processing; nonetheless their possible genetic correlations have never been investigated. In recent years the genetic basis of differences in emotions processing and emotionality have also begun to be explored [17].

Our group is interested in exploring the genetic basis of PMO construction according to the post-rationalist approach; the present work is part of a broader investigation addressing the relationships between Inward and Outward PMO and the serotoninergic system. In its framework we have already examined the possible correlations between HTR2A gene single nucleotide polymorphisms (SNPs) and the Inward/Outward PMO, but have failed to find significant data [18], [19]. In another study we applied the most advanced bioinformatics tools to select human serotonin-related HTR1A, HTR2A, HTR2C and SLC6A4 gene polymorphisms with a view to predicting which variations could give rise to biological effects, for use in future association studies in psychiatry and psychology [20], [21].

The most frequently investigated candidate gene for emotionality and personality traits is the 5-HTTLPR functional polymorphism in the promoter region of the SLC6A4 gene, which encodes the serotonin transporter [22]. The polymorphism involves a short (S) and a long (L) allelic variant that are characterized by a 44 bp deletion or insertion, respectively [23]. Detailed examination of the polymorphic region enabled Nakamura et al. to identify 10 sequence variants and to divide the allelic and S/L traditional variants into four and six types, respectively [24]. However the majority of transcriptional activity studies have been based on PCR with agarose gel electrophoresis; these investigations have shown that the S variant is associated with decreased transcriptional efficiency of the SERT promoter and reduced expression and availability of serotonin transporter (5-HTT) protein [22], [23], [24]. Initially the S allele was reported to be associated with anxiety-related traits or neuroticism [25], [26] and was thought to play a causal role in the development of affective disorder [27], [28]; however, subsequent research has suggested that it has a moderating role in the presence of environmental factors. A study of genotype-environment interactions in depression [29] involving a cohort of young men and women who had experienced multiple recent environmental adversities found that the probability of a major depressive episode and suicidality was much greater (approximately double) among individuals homozygous for the S allele than in those homozygous for the L allele. Although the gene-environment hypothesis was supported by several subsequent studies [30], [31], two extensive meta-analyses [32], [33] then failed to demonstrate an association between the serotonin transporter genotype, alone or combined with stressful life events, and an elevated risk of depression. While McGuffin et al. [34] have emphasized the methodological heterogeneity of the studies they reviewed, a more recent meta-analysis [35] based on a different, more inclusive approach, supports the hypothesis that 5-HTTLPR modulates the relationship between stress and depression. Current theoretical evolutionary models suggest that the 5-HTTLPR polymorphism might affect individual susceptibility to environmental influences rather than represent a vulnerability gene or a risk allele for the development of one or more psychiatric disorders [36], [37]. Indeed an increased attentional bias for threat stimuli [38], [39]; a greater reactivity of the amygdala to different stressors [40], [41], [42], [43], [44]; and an increased reactivity to the emotional stimuli of neural responses (amygdala, insula, anterior cingulate and ventromedial cortex) in fear acquisition have been documented among S-allele carriers [45]. Research into the relationship between the 5-HTTLPR polymorphism and differences in the neuroendocrine response to stress can provide information on the susceptibility of S-allele carriers to environmental outcomes. Even though awakening cortisol values were higher in S- than in L-allele carriers [46], [47], not all studies concluded that the former exhibit increased cortisol reactivity [34], [48], [49], [50], [51], [52], [53], and some stated the need for taking into consideration additional variables, particularly gender [48], [49], [54], [55], age [56], and other allelic variants [57]. Such greater vulnerability to external events may on the one hand underpin the increased stress reactivity of S-allele carriers, but on the other hand it may enhance their ability to grasp opportunities and to avoid potentially harmful interactions [58].

Although the results of genotype/personality studies are difficult to interpret, several papers suggest that the 5-HTTLPR genotype plays a role in susceptibility to environmental influences, contributing to a different response or resilience to stress.

This study seeks a relationship between 5-HTTLPR alleles and/or genotypes and the Inward/Outward PMO; in particular, it investigates whether individual ways of perceiving care-giver attitudes, which may involve different but consistent ways of experiencing events, may result from a predisposition.

## Materials and Methods

### Participants

We studied 124 young adult healthy Italians (mean age 35±10.8, 63 females and 58 males) without an Axis I diagnosis as evaluated with the Structured Clinical Interview for Diagnostic and Statistical Manual of Mental Disorders IV. Volunteers were recruited by advertising the study on the notice boards of the Polytechnic University of Marche (UPM) Medical School and the Health Service offices of Ancona.

The study was approved by the UPM Research Ethics Committee. All subjects gave their written informed consent to participate and authorized processing of their personal data in line with the relevant Italian regulations (Legislative Decree no. 196, 30 June 2003).

### Post-rationalist assessments

Participants' PMOs were assessed using three tools: a semi-structured post-rationalist clinical interview; the “Mini Questionnaire of Personal Organization” (MQPO), a 20-item self-rating questionnaire; and the “Post-Rationalist Projective Reactive” test (PRPR), which is the first post-rationalist projective instrument.

The clinical interview investigated the subjective manner in which each participant self-referred experience in at least two episodes involving anger and fear [14] that he deemed meaningful. Using the slow-motion (“moviola”) setting proposed by Guidano, participants were asked to recall episodes of their life and then to focus on a significant scene. They were also required to focus on the difference between “immediate experiencing” (“what” happens and “how” it happens) and “explanations” (“why” something happens) before, during and after the significant scene [11]. To do this they were asked to focus on the “how”, which "has to do with the subjective experiencing, both in terms of how it is made up, that is, its ingredients (e.g., ongoing patterns of flowing imagery; multifaceted, opposing feelings; the felt sense of self) and in terms of how it comes about, that is, what perception of events or circumstances brought it on” [2]. Participants were diagnosed for Inward/Outward PMO independently and blindly by two trained psychotherapists (B.N., E.A.) from the Psychiatric Unit the UPM Neuroscience Department.

Within the Inward and Outward general categories of identity construction the Post-rationalist approach posits four personality styles: "controller" (or "phobic" in Guidano's framework) and "detached" (or "depressive" in Guidano's framework) in the Inward group and "contextualized" (or “Psychogenic Eating Disorders” in Guidano's framework) and "principle-oriented"(or “Obsessive” in Guidano's framework) in the Outward group.

The self-report MQPO questionnaire [59] assigns participants to one of the two Inward (controller and detached) or one of the two Outward (contextualized and principle-oriented) PMOs based on the prevalence of one of the four PMO scales, whose score had to be at least 10% higher than the scores on the other scales.

The PRPR projective test [11] has been designed as a projective tool and envisages administration of 20 stimulus tables to each participant individually. It is based on the story-building technique and involves the description of subjective elements and meanings to assign participants to one of the two Inward or the two Outward PMOs. For each of the 20 stories the examiner gives points on three scales: Emotions, Reciprocity, and Intensity of Availability (hereinafter referred to as Intensity). The dichotomic Inward/Outward classification is based on scores on the Emotions and the Reciprocity scales; in particular, the Outward profile is characterized by a predominance of 3 scores (the maximum score) on the Emotions and Reciprocity scales, whereas the Inward profile is characterized by a predominance of 1 scores (the lowest score) on the Emotions and Reciprocity scales.

Participants were assigned to the Inward or the Outward group if the separate clinical evaluations of the two psychotherapists and both tests agreed on the prevalence of an Inward or an Outward PMO.

### Genotyping

**Figure 1:**
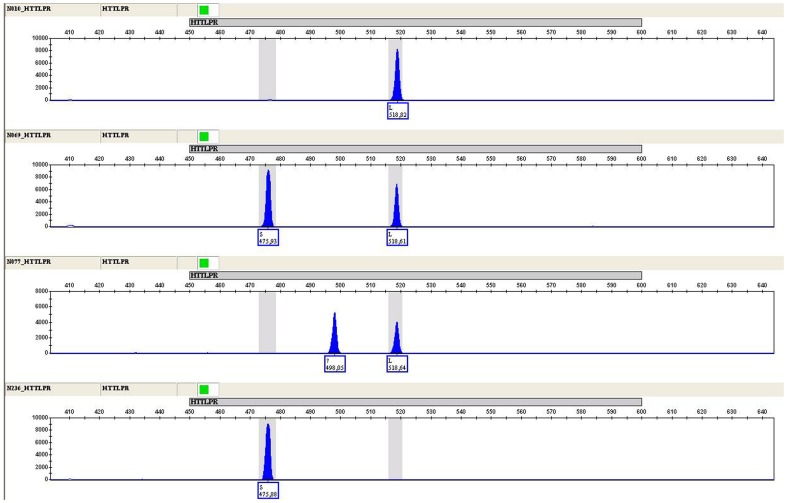
Electropherogram of the 5-HTTLPR polymorphism typed by capillary electrophoresis in four different samples. From top to bottom: L-allele homozygosity; S/L heterozygosity; L-allele heterozygosity and intermediate form; S-allele homozygosity.

Genomic DNA was extracted from buccal swabs with DNA IQ™ Reference Sample Kit 16 on the automated Maxwell™ 16 Instrument (Promega Corporation, Madison, WI, USA). The 5-HTTLPR polymorphism was genotyped by PCR [60] followed by a capillary electrophoresis discrimination assay in a 3130 Genetic Analyzer (Life Technologies, Van Allen Way Carlsbad, CA, USA). The S- and L-allele call (Figure 1) was automatically assigned by GeneMapper ID software v3.2.1 (Life Technologies, Van Allen Way Carlsbad, CA, USA).

### Statistical analysis

Hardy-Weinberg Equilibrium (HWE) was tested for the genotype frequency distribution of the 5-HTTLPR polymorphism in the whole population and separately in each Inward and Outward group using the exact test in the PowerMarker 3.25 program [61]. Associations between alleles, genotypes and phenotypes were tested by comparing allele and genotype frequency distributions within the Inward and the Outward group by the χ2 test and an exact test implemented in the PowerMarker 3.25 program.

The genotypic data were also analyzed based on two genetic models: the dominant inheritance model hypothesized that carrying the S allele increased the probability of developing an Outward PMO; the recessive inheritance model assumed that two copies of S are required to increase the probability to develop an Outward PMO. The first model involved pooling of SL and SS genotypes, the second involved pooling of SL and LL genotypes. The χ2 test and the exact test were applied and odds ratios (ORs) with 95% confidence intervals (95% CI) computed to assess the probability, conferred by each allele/genotype, of developing each PMO.

Differences in gender and age between the Inward and Outward PMO groups were analyzed with Student's t test and the χ2 test.

The serotoninergic system is held to act differently in men and women [62]. Although several studies have suggested gender differences in the interactions between the serotonin transporter gene polymorphism (5-HTTLPR) and stressful life events, and different effects on some behaviors [63], [64], [65], the topic is still controversial [66]. To avoid potential confounding effects due to gender differences between the Inward and Outward group logistic regression analysis, applied to estimate the effect of the 5-HTTLPR polymorphism on PMO, was adjusted for this demographic covariate. The analysis was performed using the PASW 17.0 program with the Inward or Outward PMO as the dependent variable and gender, alleles or genotypes as the independent variables.

For allele data, only the information regarding the minor S allele was entered into the regression model (Model 1: allelewise analysis), and was coded respectively as 1 (minor allele homozygosity, S/S); 0.5 (heterozygosity, S/L), or 0 (major allele homozygosity, L/L). In the genotype regression model two modes of inheritance were tested, Model 2 (genotype-wise recessive analysis) and Model 3 (genotype-wise dominant analysis). For recessive inheritance genotypes were coded as 1 (S/S) or 0 (S/L+L/L); for dominant inheritance they were coded as 1 (S/L+S/S) or 0 (L/L). In the regression analyses gender was the sole categorical variable.

The significance level α of all tests was 5% (p≤0.05). However, to avoid false-positive association signals, the significance level of the tests was corrected for multiple comparisons by applying a Bonferroni correction to four different comparisons: allelic, genotypic, recessive and dominant model (p = 0.05/4 = 0.0125).

Finally, a power analysis using the Episheet spreadsheet (http://www.us.oup.com/us/companion.websites/0195135547/downloads/)was carried out for the recessive model in the Inward and Outward group to establish the power of the study to detect associations.

## Results

Of the 124 volunteers, 52 were classified as having an Inward and 72 as having an Outward PMO. Concordance between the results of the two tests and between the two psychotherapists was 100%. The mean age of the Inward PMO group was 36.3±12.6 years and the mean age of the Outward PMO group was 34.1±9.3 years (*p* = 0.288, Student's t-test). Males were 56% of Inwards and 42.3% of Outwards (*p* = 0.136, χ2 test).

5-HTTLPR polymorphism analysis by capillary electrophoresis showed the S allele and/or the L allele in all participants but one. This individual had an intermediate allele ca. 20 bp shorter than the L allele (Figure 1) that was not included in the statistical analysis.

The genotype frequency distributions of the S and L polymorphisms were in HWE in the whole population (*p* = 1) as well as in Inward and in Outward subjects (*p* = 0.378 and *p* = 0.621).

The allele and genotype frequencies of the 5-HTTLPR polymorphism are listed in Table 2. Allele frequencies were significantly different (*p* = 0.036, χ2 test; *p* = 0.026; exact test) in Inwards vs. Outwards, whereas genotype frequencies were not significantly different (*p* = 0.056, χ2 test; *p* = 0.059, exact test).

**Table 2:** Comparisons of genotype and allele frequency distributions of the 5-HTTLPR polymorphism in the Inward and Outward PMO group.

	Inward		Outward			
	subjects		subjects			
Genotypes or alleles					?^2^	Exact
	N	f	N	f	*p*-value	*p*-value
S/S	5	0.096	19	0.268		
S/L	28	0.538	33	0.465	0.056	0.059
L/L	19	0.365	19	0.268		
S	38	0.365	71	0.5	**0.036**	**0.026**
L	66	0.635	71	0.5		

As regards the analysis of 5-HTTLPR genotypes based on inheritance models (Table 3), the dominant inheritance model failed to highlight significant differences between the Inward and the Outward PMO genotype (*p* = 0.246, χ2 test; *p* = 0.168, exact test; OR = 0.63, CI (95%) = 0.293–1.372), whereas significant differences were found using the recessive inheritance model (*p* = 0.0178, χ2 test; *p* = 0.0143, exact test; OR = 3.43, CI (95%) = 1.188–9.925).

**Table 3:** Comparisons of genotype frequency distributions of 5-HTTLPR polymorphism between Inward and Outward PMO groups assuming different genetic models.

	Inward subjects	Outward subjects	?^2^	Exact	OR	CI (95%)
			*p*-value	*p*-value		
Recessive model						
S/S	5	19	**0.018**	**0.014**	**3.434**	1.189–9.926
S/L+L/L	47	52				
Dominant model						
S/S+S/L	33	52	0.246	0.168	0.635	0.293–1.372
L/L	19	19				

Differences in age or gender between the Inward and the Outward PMO group were not significant (*p*≥0.05). Nevertheless logistic regression analysis, performed to establish whether the association found between the 5-HTTLPR polymorphism and PMO was attributable to differences in gender, showed that the S allele and S/S genotypes remained associated with PMO when gender was included (Table 4). Specifically, the minor S allele emerged as a risk factor for developing an Outward PMO (β = 1.243, *p* = 0.026) and the S/S genotype as a risk factor for developing an Outward PMO through a recessive mode of inheritance (β = 1.184, *p* = 0.030).

**Table 4:** Logistic regression analysis of the 5-HTTLPR polymorphism in Inward and Outward PMO subjects.

Outward subjects *vs* Inward subjects		
	β	*P*-value
Model 1: allele-wise analysis		
Alleles	**1.243**	**0.026**
Gender (F)	0.541	0.157
Model 2: genotype-wise recessive analysis		
Genotypes	**1.184**	**0.030**
Gender (F)	0.494	0.196
Model 3: genotype-wise dominant analysis		
Genotype	0.602	0.137
Gender (F)	0.557	0.140

No differences remained significant after correction for multiple testing, even though values for the recessive inheritance model (*p* = 0.0178, χ2 test; *p* = 0.0143, exact test) slightly exceeded *p*≤0.0125.

## Discussion

In this study we examined the relationship between PMO construction according to the post-rationalist approach and a functional polymorphism in the promoter region of the serotonin transporter (5-HTTLPR). We found evidence of interactions between the Inward/Outward PMO and the polymorphism, and show that such interactions are related to the greater frequency of the minor S allele and of the S/S homozygote found in Outward compared with Inward subjects (Tables 2 and 3). The difference in frequency was confirmed by logistic regression analysis, suggesting that bearing the minor S allele could increase the likelihood of developing an Outward PMO. There was no relationship with gender.

Comparison of genotype frequency between Inward and Outward subjects showed differences that were not significant. The biallelic nature of a genetic locus entails choosing between a dominant and a recessive genetic model. In the absence of data for either model [31], [67] we analyzed both, and found an association between Inward/Outward PMO and the 5-HTTLPR polymorphism solely with the recessive model. This finding was confirmed by logistic regression analysis, which showed that the S/S genotype increases the likelihood of developing the Outward PMO via a recessive mode of action. Although correction for multiple testing using the Bonferroni method did yield significant values, they were only slightly above significance for the recessive model. The present data do not therefore support a genetic basis for PMO development; nevertheless they do suggest the tendency for the short 5-HTTLPR allele to predispose to Outward PMO development.

As mentioned earlier, Outward subjects are prone to perceiving care-giver attitude (attachment) as being changeable, less predictable and depending on environmental demands or rules. They read external messages to achieve internal adequacy or normality; they are highly aware of their surroundings and of social signals, because the preliminary cognitive self-evaluation is essential to perceive emotions; they are also particularly vulnerable to negative judgments and social disconfirmation, which may induce a different perception of themselves as well as avoidant behaviors precisely in order to avoid negative outcomes [1], [2], [10], [11], [12], [13]. According to multiple lines of evidence S-allele carriers also show strong emotional arousal and increased sensitivity to environmental stimuli [31], [32], [66]. Furthermore, consistent with the differential susceptibility hypothesis, S/S individuals are particularly sensitive or vulnerable to both negative and positive environmental conditions [37], [38], [68]. It has been suggested that hyperactivity of prefrontal cortical regions and the amygdala may heighten hypervigilance in S-allele carriers [58] ensuring, under stable conditions, enhanced associative learning, cognition and social conformity. In other words, the neural circuits engaged in processing stimuli that have affective significance may be sensitized in S-allele carriers, resulting in a greater reactivity to environmental stimuli that in turn leads to negative outcomes in adversity and to potential gains in favorable environmental conditions. Some studies have shown that the attention of these individuals is elicited more by negative than by positive stimuli; however, to date the S-allele might be considered as a genetic plasticity factor in relation both to negative and positive life events [58], [69], [70], [71], [72], [81]. Outward subjects also seem to share with S-allele carriers several positive personality traits resulting from increased sensitivity to rewarding and motivating stimuli, like greater communication skills and creativity; moral judgment [73]; a greater ability to base their behavior on integration of information, e.g. in financial decisions [74]; greater social conformity through a strong inhibitory control and, of course, greater adaptability to environmental changes [58]. Therefore, the increased emotional reactivity may confer an evolutionary advantage when the environment provides social support, and seizing of opportunities and avoidance of potentially harmful interactions may facilitate social success [58]. On the other hand the S allele may correlate with greater reactivity to stressful life events [34]; moreover it is well established that particular environmental factors can predict adverse behavioral outcomes such as depression, eating disorder and post-traumatic stress disorder [31], [32], [35], [75], [76], [77], [78], [79], [80]. However, individuals homozygous for the S allele are more likely to develop psychiatric symptoms when exposed to stressful life events, but least likely to do so when exposed to positive or neutral life events [81], [82], [83], [84], thus further confirming the role of the short allele as a factor heightening susceptibility to environmental factors [36], [37].

In particular, S-allele carriers show higher reactivity to psychosocial stress [63], [85], [86], [87], and clinical evidence indicates that Outward subjects exhibit a heightened sensitivity to psychosocial stress [11], [12]. The quality of attachment seems to have a key role in amplifying or counterbalancing the tendency to read external messages conferred by the genotype [88], [89], [90]. According to a novel view of the adaptive meaning of the different personality styles [11], the increased reactivity to environmental stimuli of Outward subjects might improve coping with stress, as also evidenced by the greater stress resilience of S-allele carriers [91].

In addition, in Outward subjects emotional activations require recourse to self-evaluation cognitive schemata (i.e., guilt, sense of self-inadequacy, shame, pride, and fulfillment), which may only partially overlap and/or be shared with some personality traits (such as harm avoidance) that have been studied in relation to the S allele; however the findings of these investigations are controversial [92], [93]. In this regard, Mazzola et al. [16] found significant differences between Inward ("phobic-prone") subjects and Outward ("eating-prone") individuals on "Perspective Taking" (PT), a subtest of the Interpersonal Reactivity Index that measures the tendency to adopt the psychological point of view of others in everyday life, and on "Awareness of Bodily Processes", a subtest of the Body Perception Questionnaire. Outward subjects had higher PT scores compared with Inwards, whereas the Inward group was more likely to be aware of bodily processes than Outward subjects. Although the same study did not find significant differences between the Inward and Outward group on other personality aspects investigated by the NEO Five Factor Inventory and the Temperament and Character Inventory, the issue deserves further study and a larger sample size.

A possible limitation of our study is the small sample size, which may have influenced the power of the association analysis. For this reason we carried out a power analysis for the recessive model in the Inward and Outward group. Since the SS genotype displayed an odds ratio (OR) of 3.434 and a power of 0.93, the study does have sufficient statistical power. Another study limitations is a possible selection bias, since participants responded to a call posted on the notice boards of the UPM and of Ancona Health Services. Moreover, we did not address the A>G single-nucleotide polymorphism (SNP; rs25531), which might make L allele function similar to the S allele in presence of G rather than A. Even though a pilot study of our sample showed that this SNP was not polymorphic (unpublished data), 5-HTTLPR analysis should be revised by genotyping with a more expansive allele subdivision.

Moreover, we did not address the possible correlation between 5-HTTLPR and the PMO sub-phenotypes. For this reason, a relationship between this polymorphism and one or more PMO features cannot be ruled out.

Of course, personality development is a complex process that is difficult to investigate: many other polymorphisms should be examined, especially in the serotoninergic system, to identify further biological differences in emotional activation between Inward and Outward subjects.

In conclusion, this is one of the first studies supporting a putative genetic basis for interindividual differences in the development of Personal Meaning Organization. Specifically, our data suggest that bearing the S allele and S/S genotypes increases the likelihood of developing an Outward PMO; therefore even though our values only slightly exceed significance after correction for multiple testing, they do suggest that the S and S/S carriers (using the recessive inheritance model) will tend to be Outward subjects.

Further research is required to elucidate how attachment processes have not only a learned basis, but are also driven by behavioral genetic patterns. Our findings show how the connections between genetic and psychological approaches - not only “rationalist”, but also “post-rationalist” - can provide new working hypotheses to investigate personality style.
